# Impact of male infertility on self-esteem

**DOI:** 10.1192/j.eurpsy.2021.1458

**Published:** 2021-08-13

**Authors:** A. Zouari, F. Hammami, S. Ellouze, M. Turki, R. Jenhani, M. Chibani, R. Ghachem, S. Jallad

**Affiliations:** 1 Department Of Paychiatry B, Razi Hospital, tunis, Tunisia; 2 Unit Of Assisted Medical Procreation, Military hospital of Tunis, tunis, Tunisia; 3 Psychiatry (b), Hedi Chaker University hospital, sfax, Tunisia

**Keywords:** infertility, self-esteem, men

## Abstract

**Introduction:**

Infertile males experience considerable psychological distress, with feelings of inadequacy, marginalization, guilt and loss ofself-esteem.

**Objectives:**

Our study aims to investigate the impact of male infertility on men’s self-esteem and to study risk factorsfor low self-esteem.

**Methods:**

We conducted a cross sectional, descriptive and analytical study, including 108 infertile men who presented to the laboratory of reproductive biology and the unit of assisted medical procreation of Military Hospital of Tunis between June and September 2019. For each patient, we collected sociodemographic and clinical data. We used Rosenberg scale to assess self-esteem.

**Results:**

The average age of participants was 36.8 years. Eleven patients had a history of varicocele (10.18%) and six of them sufferedfrom associated erectile dysfunction (5.55%). Infertility was primary in most of patients (77.8%) with an average duration of 3.32 years. 25% of patients had at least one previous failed assisted reproductive attempt. Spermogram abnormalities were found in 78.7% of patients. The mean score of Rosenberg scale was 30.68±4.35. Low self-esteem was associated with older age (p=0.006), lower educational level (p=0.019) and longer duration of infertility (p=0.022). Men who had children had better self-esteem (p=0.022). No associations were found between self-esteem and erectile dysfunction or previous failed assisted reproductive technique attempt.

**Conclusions:**

Our results show that infertility reduces men’s self-esteem, especially of patients with lower educational level and longer duration of infertility. Physician dealing with infertility should be aware of these psychosocial aspects and offer help when needed.

